# Electrical and Low Frequency Noise Characterization of Graphene Chemical Sensor Devices Having Different Geometries

**DOI:** 10.3390/s22031183

**Published:** 2022-02-04

**Authors:** JongBong Nah, Frank Keith Perkins, Evgeniya H. Lock, Anindya Nath, Anthony Boyd, Rachael L. Myers-Ward, David Kurt Gaskill, Michael Osofsky, Mulpuri V. Rao

**Affiliations:** 1 Department of Electrical and Computer Engineering, George Mason University, Fairfax, VA 22030, USA; 2 U.S. Naval Research Laboratory, 4555 Overlook Ave. SW, Washington, DC 20375, USA; anthony.boyd@nrl.navy.mil (A.B.); rachael.myers-ward@nrl.navy.mil (R.L.M.-W.); kurtcapt87@verizon.net (D.K.G.); michael.osofsky@nrl.navy.mil (M.O.); 3 Global Foundries Inc., 1000 River St, Essex Junction, VT 05452, USA; anindya.nath@gmail.com; 4 Institute for Research in Electronics and Applied Physics, University of Maryland, College Park, MD 20742, USA

**Keywords:** epitaxial graphene, chemical sensor, contact resistance, low frequency noise, functionalization, ZnO nanoparticles, 1/f noise, N-ethylamino-4-azidotetrafluorobenzoate (TFPA-NH_2_), device geometry

## Abstract

Chemiresistive graphene sensors are promising for chemical sensing applications due to their simple device structure, high sensitivity, potential for miniaturization, low-cost, and fast response. In this work, we investigate the effect of (1) ZnO nanoparticle functionalization and (2) engineered defects onto graphene sensing channel on device resistance and low frequency electrical noise. The engineered defects of interest include 2D patterns of squares, stars, and circles and 1D patterns of slots parallel and transverse to the applied electric potential. The goal of this work is to determine which devices are best suited for chemical sensing applications. We find that, relative to pristine graphene devices, nanoparticle functionalization leads to reduced contact resistance but increased sheet resistance. In addition, functionalization lowers 1/*f* current noise on all but the uniform mesa device and the two devices with graphene strips parallel to carrier transport. The strongest correlations between noise and engineering defects, where normalized noise amplitude as a function of frequency *f* is described by a model of A_N_/*f*^γ^, are that γ increases with graphene area and contact area but decreases with device total perimeter, including internal features. We did not find evidence of a correlation between the scalar amplitude, A_N_, and the device channel geometries. In general, for a given device area, the least noise was observed on the least-etched device. These results will lead to an understanding of what features are needed to obtain the optimal device resistance and how to reduce the 1/*f* noise which will lead to improved sensor performance.

## 1. Introduction

Graphene has a great potential for vapor sensing applications because of its high electrical conductivity [1,2], large surface-to-volume ratio, high mobility (~200,000 cm^2^/Vs), low thermal and 1/*f* noise characteristics [3,4], and low room temperature contact resistance [5,6]. Graphene sheets (one monolayer thick) possess the remarkable quality that every atom is a surface atom and involved in carrier transport. Thus, even a single vapor adsorption event is transduced into an easily measured change in conductivity [7,8]. This property explains the single molecule detection of gas phase molecules [3]. Even though graphene is extremely sensitive, it is not selective due to its inert nature. This is remedied by functionalization with organic linker molecules, nanoparticles, biomolecules, etc., which enhances the selectivity [9,10,11,12,13].

Graphene chemical vapor sensors, fabricated as two-terminal devices exposed to ambient, operate on the principle that their electrical resistance changes in response to changes in ambient molecule concentration and, in particular, to adsorption from chemical vapors. Maintaining a low contact resistance in these devices is crucial for maximizing the relative effect of chemical-exposure-induced changes in device resistance. Published values of graphene–metal Ohmic contact resistance vary considerably depending on the fabrication approach [14,15,16,17,18,19,20,21,22]. Additionally, graphene has very low intrinsic noise, so any changes produced by defects, both intrinsic (e.g., lattice vacancies) and extrinsic (e.g., engineered holes and the associated edges in addition to the exposed edges of a finite device), as well as surface functionalization and adsorbates [23], can have a significant impact on the observed low frequency (*LF*) noise behavior. Understanding the noise behavior of electronic devices is important from an application point of view as well because this LF noise determines the smallest amount of information or signal from a device that can be detected, whether a device is functioning as an amplifier, a transducer, or merely a conveyer of information [24]. The LF (<1 Hz) spectral region is typically dominated by what is commonly referred to as 1/*f* or flicker noise, in which the power spectral density (PSD) of electrical fluctuations is inversely proportional to exponentiated frequency, *f*^γ^, with γ ranging from 0.5 to 2 but often close to 1 [24,25,26,27]. This 1/*f*^γ^ noise has been observed in many material systems [28,29]. This LF behavior is typically determined by intrinsic device or material properties, in particular dynamic changes in carrier concentration or carrier mobility associated with trap nature and density, generation-recombination centers, lattice scattering from phonons, transport scattering points associated with impurities and vacancies, and so on. Analysis of LF behavior provides insight into the physical properties of both material and devices [26,29].

In this work, we carried out electrical conductivity and LF current noise measurements at room temperature in air ambient conditions on large-area monolayer graphene devices with and without ZnO nanoparticle functionalization. Zinc oxide nanoparticles are one of the most widely investigated structures for chemical sensing due to their high thermal and chemical stability. Zinc oxide is an n-type semiconductor with a wide band gap energy of 3.37 eV. It has been reported that ZnO nanoparticles alone, and as part of a graphene composite system, have a high sensitivity towards methane, nitrogen dioxide, hydrogen, and ammonia, as well as ethanol and acetone [30,31,32]. Maximizing sensor sensitivity is of paramount importance, and in this work, we explore the relationship among device geometry resistance, and noise for pristine and functionalized devices. The investigated geometries include arrays of squares, circles, holes, and slots transverse or longitudinal to the applied electric field etched into a 0.16 mm^2^ mesa. Such a large device size is relevant to sensor applications where it is important to minimize ultra-low frequency noise. A large area device will maximize the number of charge carriers and so minimize 1/*f* noise [26], as well as ultimately minimizing the Poisson noise of adsorbates at low concentrations. Additionally, we explored the impact of contact resistance on LF noise.

## 2. Materials and Methods

Epitaxial graphene films were grown on the Si face of semi-insulating, on-axis 6H-SiC substrates by Si sublimation at high temperature in a chemical vapor deposition reactor [33]. As described elsewhere, a photoresist bi-layer method combining LOR and Shipley 1811 photoresists was used with contact printing at λ = 320 nm in two steps to achieve a clean and patterned post-fabrication graphene active region [34] and low graphene-metal contact resistance [35] after processing. The first step used oxygen plasma etching in two sequential steps of two minutes each at 30 W in order to minimize sample heating during the etch. After stripping in a 75 °C bath of N-methyl pyrrolidinone (NMP), followed by an isopropanol rinse, we applied the second photolithographic process step for patterned e-beam evaporation and lift-off in NMP to form Ti/Au (10 nm/100 nm) contacts. The films were functionalized using N-ethylamino-4-azidotetrafluorobenzoate (TFPA-NH_2_) as a chemical linker, which results in increased functionality of the graphene films without degradation of its electrical properties [36]. Then, zinc oxide nanoparticles (50–80 nm, US Research Nanomaterials Inc., Houston, TX, USA) were attached [37].

**Figure 1:**
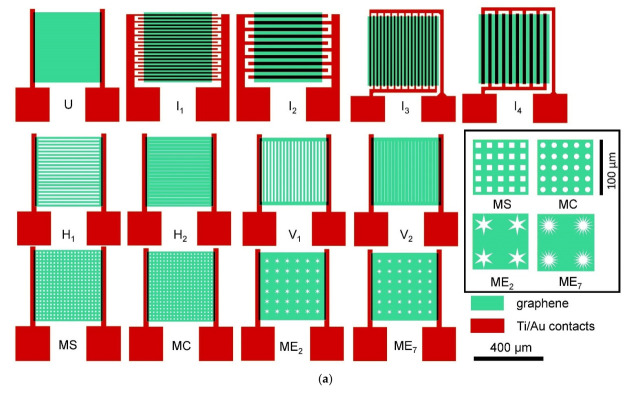

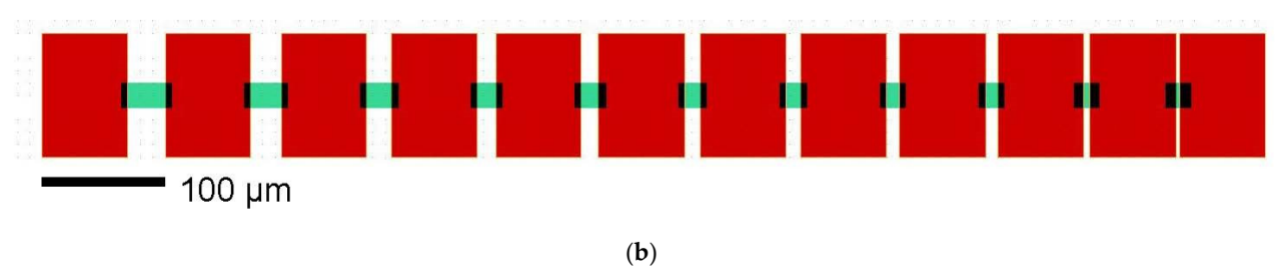
(**a**) Computer-aided design (CAD) schematic of the device designs studied here. Note that four die were printed on a chip (8 × 8 mm^2^ area). The devices are classified into four groups based on graphene film patterning: (1) unpatterned (labeled “U”) and interdigitated group (labeled “I_1_, I_2_, I_3_, I_4_”); (2) patterned with horizontal slots (labeled “H_1_, H_2_”); (3) patterned with vertical slots (labeled “V_1_, V_2_”); (4) patterned with 2D patterns (labeled “MS, MC, ME_2_, ME_7_”). Detail of the 2D patterns is shown in the inset. Descriptions are provided in Table 1. (**b**) CAD schematic of the TLM structures. The graphene mesas are 20 µm wide, the 70 µm × 100 µm Ti/Au pads overlap the graphene by 5 µm, and the uncovered lengths are 30, 25, 20, 15, 14, 13, 12, 11, 10, 5, and 3 µm.

**Figure 2:**
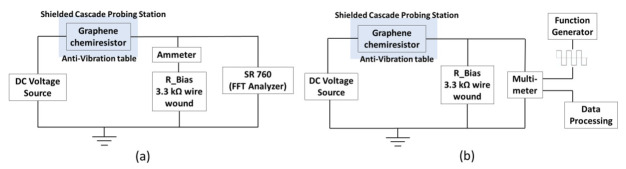
SR760 fast Fourier transform (FFT) spectrum analyzer noise measurement setup for graphene devices in a frequency range (**a**) from 0.24 Hz to 97.5 Hz and (**b**) from 0.001 Hz to 1 Hz at room temperature. A 3.3 kΩ wire wound resistor converted the induced current into a voltage for sampling either automatically by the SR760 or by an Agilent 34401A multimeter as triggered by an Agilent 33250A function generator.

**Figure 3:**
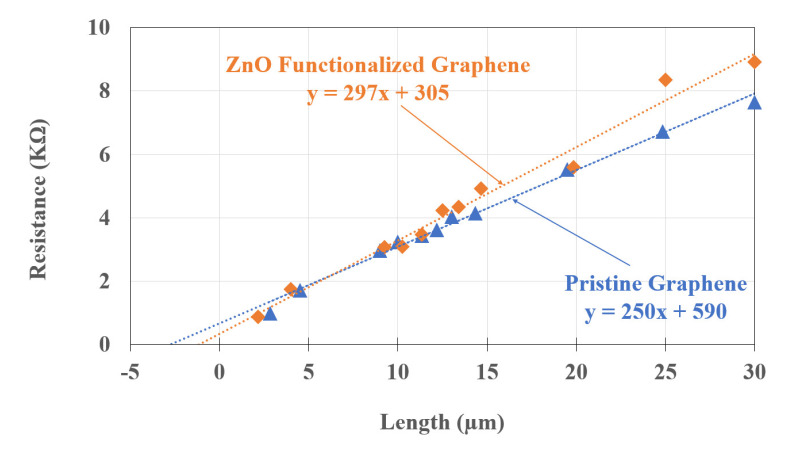
Resistance of pristine and ZnO functionalized graphene as a function of distance between metal contacts as measured after fabrication. The dotted lines are the transfer length method (TLM) linear fits.

**Figure 4:**
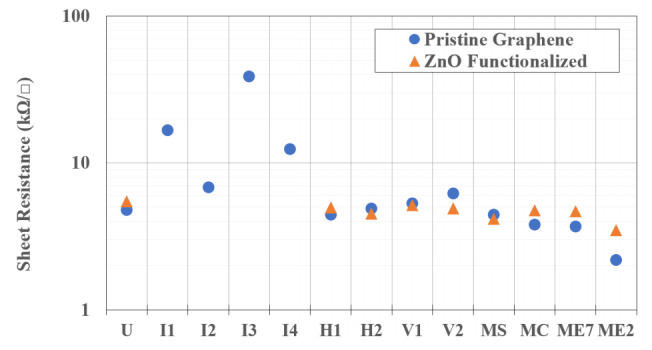
Sheet resistance, accounting for internal etched features, of different geometries on pristine and ZnO functionalized graphene as calculated from data extracted from TLM measurements and graphene features.

Graphene devices were fabricated on 8 × 8 mm^2^ SiC chips bearing 4 die, each die with 13 sensor devices (schematic shown in Figure 1a) and 2 to 4 transfer length method (TLM) structures (Figure 1b) for measuring contact resistance. The various chemiresistive sensor device structures, all built on a common 400 × 400 µm^2^ graphene mesa, can be classified into four groups. The devices, except for the interdigitated group, had the same channel length of 380 µm and varying channel widths. Group 1 consisted of an unpatterned graphene device (U) and devices having interdigitated electrodes, with 10 µm (I_1_, I_3_) and 20 µm (I_2_, I_4_) channel lengths and corresponding channel widths of 3600 µm and 7600 µm, respectively. They were printed in either of two orthogonal orientations in order to explore directional dependence of conductivity in the epitaxial graphene. Group 2 consisted of patterned devices with slots aligned with charge transport (henceforth “horizontal” and identified as H_1_, H_2_). Group 3 consisted of patterned devices with slots transverse to charge transport (henceforth “vertical” and identified as V_1_, V_2_). Group 4 consisted of patterned devices with regular 2D hole patterns including 16 point (ME_7_) and 6 point stars (ME_2_), 10 × 10 µm^2^ squares (MS), and 10 µm dia. circles (MC). The TLM structures were fabricated with contact separations from 3 to 30 µm and a uniform width of 20 µm. The Ti/Au contacts were 70 × 100 µm^2^ rectangles that overlapped 5 µm of the graphene films, thus adding a metal–graphene edge junction of nominally 30 µm at each contact. See Table 1 for a detailed description.

A Keithley 236 source measurement unit (SMU) supplying 1 VDC was used to measure device resistance. Two configurations were used for room temperature low frequency noise measurements, from 0.24 Hz to 97.5 Hz and from 0.001 Hz to 1 Hz, as shown in Figure 2a,b, respectively. We measured the current noise of the devices by measuring the voltage across a 3.3 kΩ wire wound precision resistor in series with the sensor device using a spectrum analyzer with an input impedance of 1 MΩ‖15 pF. A Hann window was applied to each frame prior to the application of a fast Fourier transform (FFT). The resulting frequency data was averaged for at least 5 such scans.

## 3. Results and Discussion

### 3.1. Resistance Measurements

In this section, we report the resistance properties of pristine and ZnO functionalized graphene films and devices. First, the contact resistance (*R_c_*), sheet resistance (*R_sh_*), and contact resistivity (*ρ*_c_) of the graphene films were determined from the TLM structures described above. Next, we measured the devices’ total resistance. Finally, we calculated the effective width, contact area, contact resistance, and sheet resistance for the device structures.

In general, the resistance (R) of any material is given by *R* = *ρL*/*A*, where *ρ* is the bulk resistivity, *L* is the length, and *A* is the cross-sectional area (width *W* × thickness *t*) of the material in a plane normal to the direction of current flow. For graphene films, we assume that the films are of uniform thickness, so that the cross-section is determined entirely by the effective device width. For the group 2, 3, and 4 devices (Figure 1, Table 1), the width at the constrictions should dominate the overall measured resistance. A more complete picture of device resistance also considers contact resistance and the effect of the depletion length at the contacts, especially in comparison to the overall channel length. The interdigitated structures I_1_ through I_4_ offer an opportunity to observe directional dependence of electrical conduction in graphene [38,39,40]. Transport in I_3_ and I_4_ is in the same average direction as in the devices other than I_1_ and I_2_: U, Group 2, Group 3, and Group 4.

We present in Figure 3 the total resistance (*R*_T_) of the TLM structures plotted against the varying graphene channel lengths for both pristine and functionalized graphene films. The slope of the linear fit in Figure 3 gives the channel width a normalized value (*R_sh_*/*W*) of the graphene sheet resistance. The contact resistance, *R_c_*, is extracted from the extrapolated resistance at zero channel length, 2 × *R_c_*. The contact resistivity is determined from the equation *ρ*_c_ = *R_c_* × *W* × *L_T_*, where *L_T_* is the transfer length, the average distance that an electron travels in the material beneath the contact before it flows up into the contact. The transfer length, *L_T_*, is obtained from the extrapolated length at zero resistance (−2L_T_ = *x*-intercept) [41].

The calculated *R_c_*, *R_sh_*, L_T_, and *ρ*_c_ for pristine and functionalized graphene films are shown in Table 2. There is a substantial decrease in contact resistance (factor of two) and in contact resistivity (factor of 3.5) and a moderate increase in sheet resistance of the functionalized films relative to that of the pristine graphene. This change in *R_sh_* is consistent with a previous investigation [36], which also found a substantial decrease in carrier sheet concentration to about 4 × 10^12^ cm^−2^ and an increase in carrier mobility of functionalized films relative to pristine. Following the approach of Nath, et al. [35] we apply the Landauer–Büttiker model for the conductance of a one-dimensional wire to represent the graphene–metal contact resistance as:

(1) RC=1Thπ124e2n12

where *T* is the carrier transmission probability, *h* is Planck’s constant, *n* is the sheet carrier concentration, and *e* is the electron charge underneath the metal [35,42,43]. For perfect quantum limited contacts, one assumes T = 1, and this gives a lower bound to contact resistance, assuming *n =* 4 × 10^12^ cm^−2^, of about 57 Ω-µm. In our case, the functionalized sheet resistance has increased by 20% compared to pristine graphene. Assuming the previously observed drop in sheet carrier concentration after functionalization to have also occurred here, then the nearly two-fold reduction in contact resistance must be due to increased T, which could be explained by changes in the film work function induced by functionalization that lowers the interfacial barrier height.

Next, we measured the R_T_ of the pristine and functionalized graphene devices (Table 3). In general, the R_T_ values of pristine and functionalized devices are very similar with the exception of the MC, ME_7_, and ME_2_ devices from Group 4. We found that the structure group, and the specific features within the group, have a much stronger effect on the measured values. In general, group 3 (V_1_, V_2_) had the highest R_T_ values, and group 1 (I_1_–I_4_) the lowest. Group 1′s unpatterned device U, group 2, and group 4 had similar total R_T_ values 4.5–9 kΩ. The relatively smaller normalized resistance of the Group 4 devices is not surprising, as some current spreading in the regions between the etched features is to be expected.

Finally, we calculated the effective contact area, the device contact resistance *R_C_*, and the device sheet resistance *R_sh_* of pristine and functionalized graphene device structures for the different device geometries. The extrinsic contact resistance R_c_ (not the intrinsic resistivity) for the actual sensor device is calculated from geometry and values of L_T_ and ρ_c_ calculated from the TLM structures, estimating *R_c_* for each sensor device from ρ_c_ as derived from the TLM data, the overlapping contact width *W_c_*, and the calculated transfer length *L_T_* from Table 2 using the relation:

(2) $$R_{C}=\frac{\mathrm{Contact}\;\mathrm{Resistivity}}{\mathrm{Contact}\;\mathrm{Area}}=\frac{\rho_{c}}{W_{C}\cdot L_{T}}.$$

Sheet resistance is calculated here simply from *R_sh_ = (R_T_* − *2R_C_)* × *W_ch_/L*, where *W_ch_* is the effective channel width after accounting for etched features and neglecting lower resistance (i.e., wider) sections (Table 1). The resulting values are plotted in Figure 4, and given Table 4. The *R_sh_* of devices I_1_, I_2_, I_3_, and I_4_ is quite large compared to the expected value obtained from the TLM structures given in Table 2, as well as that of the other devices. Since the typical depletion width in graphene, which would decrease the effective channel length, is quite small, of order 100 nm or less, further work is needed to understand the inconsistency observed here. A desired condition for sensor applications, namely, the relative relationship *R_c_* << *R_T_*, is realized for all but the interdigitated device geometries in this study.

The effective channel widths (*W*_eff_) can be calculated using the relation *W*_eff_ = (*R_sh_* × *L*)/*R_ch_*, where *R_sh_* is the sheet resistance calculated from the TLM data (Table 2), *L* is the graphene channel length, and *R_ch_* is the channel resistance, *R_T_* − *R_c_*. For comparison, the calculated *W*_eff_ and width values for pristine and functionalized graphene are shown in Table 5.

The *W*_eff_ calculations are not applied to the interdigitated devices due to the uncertainty in the effective channel length, which is much smaller than the mask channel length L, as explained above. A defect, such as the termination of a crystalline lattice structure at an interface, can contribute a mobile charge and change the number of carriers. In normal materials, this can increase or decrease the conductivity depending on the type of the added carrier, majority, or minority. Graphene is ambipolar, so the addition of more carriers of either type simply increases the conductivity. A defect can also create a fixed dipole which can scatter charges, lowering mobility and, hence, conductivity if it is in the path of transport. Charges created at defect sites will diffuse away from areas of high concentration (where the defects are) to areas of low concentration (normal film) but usually leave a fixed charge behind which may be partially screened. In the devices discussed here, defects are present from both the structures etched into the graphene, as well as when the organic linkers and nanoparticles are added during the functionalization process. The precise nature and impact of each will be addressed in future work.

### 3.2. Low Frequency Noise in Graphene Devices

The current noise of our devices was measured by sampling the voltage developed across a resistor in series with the sensor device when a 1V DC bias was applied across the pair. A wire-wound resistor was used (rather than metal film, etc.) to reduce the contribution to the measured noise from that component [44]. The value of the resistor, 3.28 kΩ, was selected to match the average resistance value of all of the devices. In order to obtain a power spectrum *S*^2^(*f*_n_) of the device noise, we note that the discrete Fourier transform of a set of *N* voltage samples *V_d,k_*, *k* = 0, 1, …, *N* − 1, collected across the device at time intervals of width Δ, where the device is in series with the resistance *R* dividing a voltage *V*:

(3) $$S(f_{n})\approx \Delta \sum_{K=0}^{N-1}(V_{d,k})e^{\frac{2\pi ikn}{N}}=\;\Delta \sum_{K=0}^{N-1}(1-V_{R,k})e^{\frac{2\pi ikn}{N}}=\Delta \left[\sum_{K=0}^{N-1}e^{\frac{2\pi ikn}{N}}-\sum_{K=0}^{N-1}V_{R,k}e^{\frac{2\pi ikn}{N}}\right]$$

is equivalent to sampling the voltage across the device *V_d,n_* because the first term in the right-hand expression vanishes due to orthogonality, and the negative sign on the second term vanishes when the term is squared during subsequent processing, leaving the following:

(4) $$S(f_{n})\approx \Delta \sum_{K=0}^{N-1}V_{R,K}e^{2\pi ikn/N}$$

**Figure 5:**
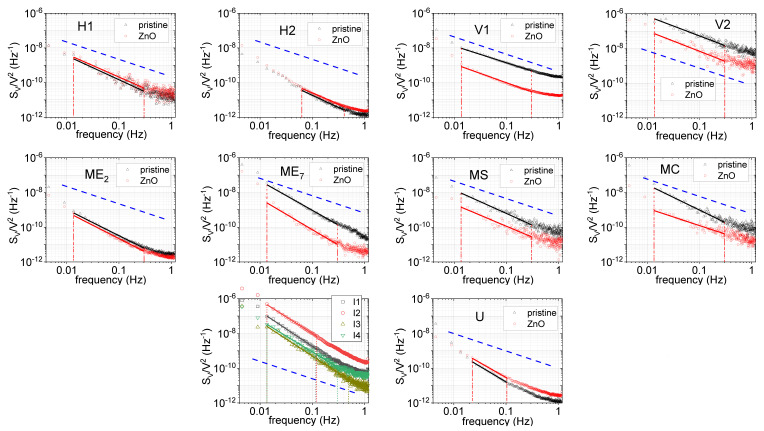
Normalized (*S_V_*/*V*^2^) noise data, plotted vs. frequency and for each device: Pristine graphene devices are plotted with black triangles, functionalized devices are plotted with red circles. The four interdigitated devices of pristine graphene I_1_–I_4_ are also shown. A linear fit to a portion of the power spectrum, and the frequency range over which it was calculated, is shown for each data set. A representative 1/*f* line is also shown on each graph as a blue dashed line; the vertical placement is arbitrary, with no significance.

The voltage data were obtained by sampling at 2.31 Hz over 665 s durations. Work not reported here established a corner frequency of 1–10 Hz, and there was generally no significant power or signal at higher frequencies to be aliased into this frequency range. A Hann window was applied to each frame prior to the transform, and the resulting frequency data between 0.00451 and 1.15 Hz were averaged for at least 5 such scans. After normalizing the FFT of each device response by the average voltage across the device, the results are plotted and shown by device in Figure 5 along with a linear fit of log *S_V_*/*V*^2^ vs. *f* generally between 0.015 and 0.2 Hz. The observed noise is low compared to other published work [45,46,47,48,49]. However, the consideration of an argument of Snow et al. suggests that our observed noise attenuation could be attributed to the increased device size [50].

Low frequency noise in graphene under ambient conditions has been attributed to multiple sources, including slow traps, generation/recombination (GR) centers, scattering from impurities, and dynamic changes in the scattering cross-section, presumably due to the chaotic impact on dipole screening of the constrained motion of charge carriers in the 2D film [26]. In order to analyze the performance of graphene-based devices, previous studies [24,25] have used the following empirical expression [45] to quantitatively describe the magnitude of the low frequency noise:

(5) $$\frac{S_{V}}{V^{2}}=\frac{S_{I}}{I^{2}}=\frac{A_{N}}{f^{\gamma }}$$

where *f* is the frequency, γ is the frequency scaling exponent, and A_N_ is related to the Hooge parameter α_H_ through *A_N_* = *Nα_H_*, where *N = n + p* [28]. The amplitude *A_N_* is a scalar measure of the 1/*f* noise level and generally reflects the quality of a material or a device, depending on the number of charge carriers and extrinsic parameters such as device channel area and structural and chemical condition of the material: a higher value of *A_N_* usually corresponds to a lower quality device. By comparing devices of similar area as fabricated from a common material with a common process, we can assume the number of carriers to be roughly comparable across the devices as well.

The *A_N_* and γ values for all devices were calculated by fits to linear portions of the data (generally between 0.01 and 0.5 Hz) shown in Figure 5. The objective was not to obtain a rigorously valid exponent, but rather to gain a qualitative sense of the low-frequency noise in the broadest spectral range with minimal sensitivity to narrow band features or higher frequency components (Table 6). The wide range of variability in γ is unexpected, indicative of the wide range of possible effects which contribute to noise in these devices. Most researchers reported variability in γ for monolayer graphene devices but with values near 1 [46,47,48,49]. The high values observed here for the largest, unmodified film devices U and I_1_–I_4_ indicate a large number of slow processes of duration longer than 1/2π*f* for frequencies below 1 Hz. The large size of these devices and the extended data acquisition to low frequencies may allow other processes to occur and be observed that have not previously been considered. For comparison of our observed noise to other published work, e.g., Rumyantsev et al. [4], we extrapolate the 1/*f* fit to 10 Hz, where our data are dominated by Johnson noise, and multiply the predicted value of S_V_/V^2^ by device length and effective width from Table 1 and Table 5 to obtain the values given in Table 6. In the work of Rumyantsev et al., a similar analysis of multiple, albeit considerably smaller, devices fabricated from exfoliated graphene under a controlled back-gate bias of 0 V concluded that area-normalized noise at 10 Hz fell between 1 × 10^−8^ and 1 × 10^−7^ µm^2^/Hz. In comparison, we find a generally consistent and systematic variation: Group 1 devices exhibit greatly reduced noise, Group 2 devices exhibit marginally reduced noise, Group 3 devices exhibit increased noise, and Group 4 devices exhibit a wide range of noise levels, overlapping the range of Rumyantsev et al.

To investigate which device feature influences noise the most, we studied the dependence of 1/*f* noise on device geometry, functionalization, effective graphene area, metal contact area, and mesa etched graphene perimeter. The areas and perimeters (internal and external) of graphene mesas measured after etching and metal contact areas were calculated for the geometries shown in Figure 1 and described in Table 1. From an inspection of Figure 5, it is evident that functionalization generally lowers noise in all but the H_1_, H_2_, and U devices. These three are also the quietest devices, implying that the factors contributing to noise, and passivated by functionalization, are not present in certain devices (H_1_, H_2_, and U) but exist in others, specifically V_1_, V_2_, MC, MS, ME_2_, and ME_7_. Consideration of these device geometries suggests that while a single trap/excitation, generation/recombination, adsorption/desorption, etc., event can simultaneously induce changes in mobility and carrier concentration δ*µ_n_*, δ*µ_p_*, δ*n*, and δ*p* equivalently in the H_1_, H_2_, and U devices, such changes are *not* equivalent in the V_1_, V_2_, MC, MS, ME_2_, and ME_7_ devices, where considerable graphene is spatially remote from the primary transport paths. Thus, the direct effect on mobility through the regions defined as direct paths between electrodes from events outside those paths is minimal, while generated charges can easily diffuse into the areas of current flow where drift mobility (and, thus, scattering events) are significant. The ME_2_ is somewhat exceptional. However, the sparser hole array relative to the MS and MC devices and reduced internal perimeter relative to the low field, high carrier concentration area as compared to the ME_7_ device may explain the observed results. These results suggest a rather complex relationship between mobility and carrier concentration.

**Figure 6:**
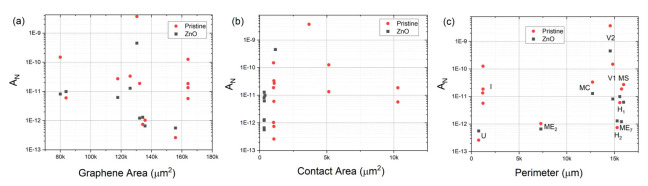
Noise scalar *A_N_* plotted against (**a**) graphene area, (**b**) contact area, and (**c**) total perimeter for both pristine and ZnO functionalized graphene devices.

**Figure 7:**
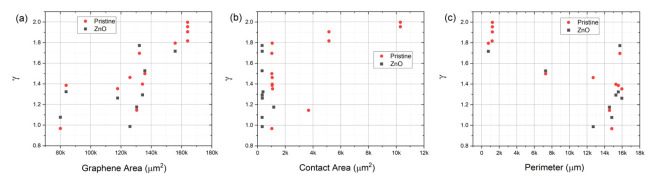
Frequency exponent γ plotted against (**a**) graphene area, (**b**) contact area, and (**c**) total perimeter for both pristine and ZnO functionalized graphene devices.

In an effort to look for correlations between measurable parameters of graphene area, contact area, active device perimeter, and noise parameterization terms *A_N_* and γ, we present plots of these in Figure 6 (*A_N_*) and Figure 7 (γ) for the two sets of devices, pristine and functionalized graphene. The graphene area and perimeter for each device was calculated from the mask data. The contact area was calculated from the width of metal contacts and the transfer length presented in Table 2. There is no strong evidence for correlation with the device active (graphene) area or the contact area of the noise scalar A_N_. If one neglects the four interdigitated devices, where the relatively high currents and negligible perimeter relative to area suggests different relevant physical phenomena, there may be a correlation between perimeter and noise scalar *A_N_*. We do see strong correlations between the device area and γ and the contact area and γ and an inverse correlation between the perimeter and γ. This strongly suggests that different mechanisms exist in the generation of noise in the different regions of the film, which can be used to improve sensor design.

Ultimately, the question of which is the optimal sensor design is still unsettled. Although in this work we have addressed the relationship of design to intrinsic device noise, we have not fully examined the relationship of design to extrinsic signal, i.e., chemiresistive response. It may be the case that the noisiest device geometries are also the most sensitive. In any case, a characteristic and reproducible response behavior defined as change in conductance should be achieved rapidly. Furthermore, the design of the sensor should strongly favor detecting perturbations in conductivity induced by adsorption of target species over others. Perturbations due to background or benign chemicals, which are also considered to be noise, should be minimized. Finally, nanoparticles other than 50–80 nm dia. ZnO may have different impacts on noise and sensor response. These factors will be explored in future work.

## 4. Conclusions

We have carried out measurements of resistance and low-frequency noise in graphene devices to determine the effects of ZnO nanoparticles functionalization and the engineered defects of the graphene channel. The goal of our work was to understand which device is best suited for chemiresistive sensing applications. For comparison purposes, all of the devices studied had the same graphene mesa area, but with different defects patterns of interdigitation or etched internal holes. These hole patterns included arrays of coarse and fine long slots etched perpendicular and parallel to the applied field, and two-dimensional arrays of squares, circles, and few and many pointed stars. We find that functionalization generally lowers noise, with the exceptions being the three quietest devices (H_1_, H_2_, and U), implying that the factors contributing to noise, and passivated by functionalization, are not present in some geometries but exist in others. The resistance measurements showed that devices with long etched stripes orthogonal to the direction of the applied electric field have the highest resistance, and short and wide channel interdigitated devices have the lowest resistance for both pristine and ZnO-functionalized graphene. The graphene–metal Ohmic contact resistances (*R_C_*) demonstrate that ZnO-functionalized graphene has lower contact resistance, but higher graphene sheet resistance (*R_sh_*) compared to the pristine graphene. There is no strong evidence for a correlation between the scalar noise power and actual graphene channel area, contact area, and total perimeter (including the internal etched hole perimeters). However, there is a strong direct correlation between noise frequency dependence and graphene area and contact area. Furthermore, there is an inverse correlation between noise frequency dependence and perimeter. This work highlights that the electrical and low frequency noise measurements are critical for the selection of appropriate device structure in graphene/ZnO chemical sensors.

## Figures and Tables

**Table 1:** Detailed description of the four graphene chemiresistive device group structures shown in Figure 1. The terms “horizontal” and “vertical” refer to the graphene pattern with respect to the direction of charge transport, parallel or perpendicular, respectively. “Constricted channel width” refers to the minimum graphene channel width after etching, i.e., overall film width less total hole cross section.

Channel Structure Type	Device Designation	Channel Length, µm	Channel Width, µm (Constricted)	Detailed Description
Group 1: Unpatterned	U	380	410	Horizontal transport
	I_1_	10	7600	Vertical transport
	I_2_	20	3600	
	I_3_	10	7600	Horizontal transport
	I_4_	20	3600	
Group 2: Horizontal Slots	H_1_	380	210	21 ea. 10 µm strips
	H_2_	380	350	20 ea. 17 µm strips, 2 ea. 5 µm strips
Group 3: Vertical Slots	V_1_	380	40	2 ea. 20 µm wide strips at either end of set of slots, strips 360 µm long, 10 µm and 17 µm wide, respectively
	V_2_	380	40	
Group 4: 2D Patterns	MS	380	220	10 × 10 µm^2^ squares on regular 20 µm pitch grid
	MC	380	210	10 µm dia. Circles on regular 20 µm pitch grid
	ME_7_	380	160	416 µm^2^ area, 425 µm coded perimeter, on regular 60 µm pitch grid
	ME_2_	380	160	480 µm^2^ area, 180 µm coded perimeter, on regular 60 µm pitch grid

**Table 2:** Electrical characteristics of pristine and functionalized graphene films with evaporated Ti/Au contacts calculated using the TLM data in Figure 3 (contact width = 20 µm, length = 5 µm). Data from ref [16] refers to CVD graphene with Al/Cr/Au contacts, measurements from TLM structures. Data from ref [35] refers to annealed graphene, measurements from TLM structures.

Parameter	Pristine	Functionalized	Ref [16]	Ref [35]
*R*_c_, Ω-µm	5900 ± 800	3050 ± 1800	1497	1075 ± 285
*R_sh_*, kΩ/□	5.0 ± 0.1	5.9 ± 0.3	0.952	0.84
*L*_T_, µm	1.2 ± 0.2	0.53 ± 0.1	1.57	1.28
ρ_c_, Ω-cm^2^	7.2 × 10^−5^ ± 2.2 × 10^−5^	2.2 × 10^−5^ ± 2.5 × 10^−5^	2.35 × 10^−5^	1.38 × 10^−5^

**Table 3:** The average device total resistance values (R_T_) for pristine and ZnO functionalized graphene device structures (open cells indicate devices not studied).

Groups	Device Designation	Total Resistance R_T_ (kΩ)	
		Pristine Graphene	ZnO Functionalized
Group 1	U	4.49	5.10
	I_1_	0.03 ± 0.003	
	I_2_	0.05 ± 0.004	
	I_3_	0.06 ± 0.002	
	I_4_	0.08 ± 0.007	
Group 2	H_1_	8.14 ± 1.46	9.07
	H_2_	5.36 ± 1.80	4.93
Group 3	V_1_	50.5 ± 15.4	48.9 ± 3.69
	V_2_	59.1 ± 19.7	46.6 ± 1.58
Group 4	MS	7.73 ± 2.51	7.24
	MC	6.95 ± 1.89	8.63 ± 0.18
	ME_7_	8.84 ± 1.82	11.2 ± 1.26
	ME_2_	5.27 ± 0.63	8.31 ± 1.90

**Table 4:** Effective contact area calculated from the device channel width and film contact transfer length *L_T_* as derived from TLM data, contact resistance (*R_C_*), and calculated sheet resistance (*R_sh_*) of pristine and functionalized graphene device structures after correcting for constricted width values of the different device geometries (open cells indicate devices not studied).

Group	Device Designation	Pristine Graphene			Functionalized Graphene		
		Effective Contact Area (µm^2^)	Contact Resistance *R_C_* (kΩ)	Sheet Resistance *R_sh_* (kΩ/□)	Effective Contact Area (µm^2^)	Contact Resistance *R_C_* (kΩ)	Sheet Resistance *R_sh_* (kΩ/□)
Group 1	U	492	0.03	4.8	217	0.02	5.5
	I_1_	4800	0.003	16.7	2120		
	I_2_	2400	0.006	6.8	1060		
	I_3_	4800	0.003	38.8	2120		
	I_4_	2400	0.006	12.4	1060		
Group 2	H_1_	492	0.03	4.5	217	0.02	5.0
	H_2_	492	0.03	4.9	217	0.02	4.5
Group 3	V_1_	480	0.03	5.3	212	0.021	5.1
	V_2_	480	0.03	6.2	212	0.021	4.9
Group 4	MS	492	0.03	4.4	217	0.02	4.2
	MC	492	0.03	3.8	217	0.02	4.7
	ME_7_	480	0.03	3.7	212	0.021	4.7
	ME_2_	480	0.03	2.2	212	0.021	3.5

**Table 5:** Summary of coded width and effective width for pristine and ZnO functionalized graphene device structures.

			Effective Width (µm)	
Group	Device	Coded Width (µm)	Pristine Graphene Films	ZnO Functionalized Graphene Films
Group 1	U	410	387	389
Group 2	H_1_	210	211	200
	H_2_	350	323	373
Group 3	V_1_	40	34	37
	V_2_	40	29	40
Group 4	MS	220	222	256
	MC	210	248	214
	ME_7_	160	194	68
	ME_2_	160	329	94

**Table 6:** The 1/*f* current noise spectra parameters (Hooge and gamma) for pristine and functionalized graphene (open slots indicate devices missing (printing flaws) due to processing issues) and channel-area normalized noise from an extrapolation to 10 Hz. For comparison, previous work of Rumyatsev et al. [4] reported a channel-area normalized noise range between 1 × 10^−8^ and 1 × 10^−7^ µm^2^/Hz.

		*A_N_*		γ		*S_V_*/V^2^ · L · W_eff._ @ 10 Hz (µm^2^/Hz)	
Group	Device	Pristine	Functionalized	Pristine	Functionalized	Pristine	Functionalized
Group 1	U	2.61 × 10^−13^	5.59 × 10^−13^	1.8	1.7	5.96 × 10^−10^	1.61 × 10^−9^
	I_1_	1.86 × 10^−11^		2		3.68 × 10^−9^	
	I_2_	1.26 × 10^−10^		1.9		6.57 × 10^−8^	
	I_3_	5.72 × 10^−12^		2		4.91 × 10^−10^	
	I_4_	1.35 × 10^−11^		1.8		5.14 × 10^−9^	
Group 2	H_1_	6.02 × 10^−12^	9.92 × 10^−12^	1.4	1.3	1.90 × 10^−8^	3.94 × 10^−8^
	H_2_	7.46 × 10^−13^	1.30 × 10^−12^	1.4	1.3	3.58 × 10^−9^	7.85 × 10^−9^
Group 3	V_1_	1.49 × 10^−10^	8.18 × 10^−12^	1	1.1	1.90 × 10^−7^	8.27 × 10^−9^
	V_2_	3.71 × 10^−9^	4.51 × 10^−10^	1.1	1.2	3.22 × 10^−6^	3.11 × 10^−7^
Group 4	MS	2.73 × 10^−11^	6.22 × 10^−12^	1.4	1.3	9.07 × 10^−8^	2.60 × 10−08
	MC	3.34 × 10^−11^	1.29 × 10^−11^	1.5	1	9.81 × 10^−8^	1.20 × 10^−7^
	ME_7_	1.88 × 10^−11^	1.22 × 10^−12^	1.7	1.8	2.74 × 10^−8^	1.41 × 10^−9^
	ME_2_	1.04 × 10^−12^	6.60 × 10^−13^	1.5	1.5	4.03 × 10^−9^	2.56 × 10^−9^

## Data Availability

Not applicable.
